# Estimating the contribution of Greenland Halibut (*Reinhardtius hippoglossoides*) stocks to nurseries by means of genotyping‐by‐sequencing: Sex and time matter

**DOI:** 10.1111/eva.12979

**Published:** 2020-05-20

**Authors:** Emilie Carrier, Anne‐Laure Ferchaud, Eric Normandeau, Pascal Sirois, Louis Bernatchez

**Affiliations:** ^1^ Institut de biologie intégrative et des systèmes (IBIS) Université Laval Québec City QC Canada; ^2^ Département des sciences fondamentales Université du Québec à Chicoutimi Chicoutimi QC Canada

**Keywords:** Greenland Halibut, management, marine genomics, population assignment, stock identification, temporal stability

## Abstract

Identification of stocks and quantification of their relative contribution to recruitment are major objectives toward improving the management and conservation of marine exploited species. Next‐generation sequencing allows for thousands of genomic markers to be analyzed, which provides the resolution needed to address these questions in marine species with weakly differentiated populations. Greenland Halibut (*Reinhardtius hippoglossoides*) is one of the most important exploited demersal species throughout the North Atlantic, in particular in the Gulf of St. Lawrence, Canada. There, two nurseries are known, the St. Lawrence Estuary and the northern Anticosti Island, but their contribution to the renewal of stocks remains unknown. The goals of this study were (a) to document the genetic structure and (b) to estimate the contribution of the different identified breeding stocks to nurseries. We sampled 100 juveniles per nursery and 50 adults from seven sites ranging from Saguenay Fjord to offshore Newfoundland, with some sites sampled over two consecutive years in order to evaluate the temporal stability of the contribution. Our results show that after removing sex‐linked markers, the Estuary/Gulf of St. Lawrence represents a single stock which is genetically distinct from the Atlantic around Newfoundland (*F*_ST_ = 0.00146, *p*‐value = .001). Population assignment showed that recruitment in both nurseries is predominantly associated with the St. Lawrence stock. However, we found that the relative contribution of both stocks to the nurseries is temporally variable with 1% contribution of the Newfoundland stock one year but up to 33% for the second year, which may be caused by year‐to‐year variation in larval transport into the Gulf of St. Lawrence. This study serves as a model for the identification of stocks for fisheries resources in a context where few barriers to dispersal occur, in addition to demonstrating the importance of considering sex‐linked markers and temporal replicates in studies of population genomics.

## 1 INTRODUCTION

The use of genomic tools for conservation and management offers the opportunity to better understand the dynamics and demography of natural populations (Manel, Gaggiotti, & Waples, 2005). Population genomics allows answering conservation issues, such as assessing reproductive success (Ford, Pearsons, & Murdoch, 2015), testing for local adaptation (Grummer et al., 2019; O'Malley, Camara, & Banks, 2007), documenting migratory patterns of exploited and/or threatened populations (Larson et al., 2013), and defining management units (Bernatchez et al., 2017; Palumbi, 2004). In the case of marine species, the lack of statistical power is a main issue when trying to define stocks of exploited species, where exchange of genetic material is high due to minimal dispersal constraints and differentiation between stocks is further limited because of large effective population sizes (Waples, Punt, & Cope, 2008). However, the improvement of next‐generation sequencing since the mid‐2000s has allowed the definition of subtle differentiations at fine local scales in order to delineate populations (i.e., stocks) by making it possible to genotype thousands of markers on hundreds of individuals in a relatively short time (Metzker, 2010), as exemplified by recent studies of various marine organisms such as the American lobster (*Homarus americanus*; Benestan et al., 2015), the giant California sea cucumber (*Parastichopus californicus*; Xuereb, Kimber, Curtis, Bernatchez, & Fortin, 2018), or the Atlantic cod (*Gadus morhua*; Barth et al., 2017), to name a few.

Nevertheless, increasing the number of markers is not always sufficient to resolve population structure for species with large populations and/or high connectivity such as marine species (Gagnaire et al., 2015). For reduced‐representation sequencing data, it is known that filtering parameters have a major impact on results (Puebla et al., 2014; Rodriguez‐Ezpeleta et al., 2016, 2017; Shafer et al., 2017). For example, as shown by Gagnaire et al. (2015), removing SNPs under directional selection from assessment of migratory pattern and connectivity of marine species populations might result in elimination of informative loci with high *F*_ST_ between “real” populations and thus creates false‐negative interpretation. Also, Roesti, Hendry, Salzburger, and Berner (2012) showed that the outcome of population genomics studies can be systematically biased if markers with a low minor allele frequency are included in the analysis. The reason is that these “uninformative” polymorphisms lack the adequate potential to capture signatures of drift and hitchhiking. Sex‐linked markers, which consist of genomic markers located in genomic regions associated with sex determinism, may also influence population structure results. As shown in Benestan et al. (2017), removing SNPs linked to sex resulted in a lower and more realistic estimation of differentiation for the anadromous species Arctic charr (*Salvelinus alpinus*) and in American lobster (*Homarus americanus*). More recently, a study of stock assessment in deacon rockfish (*Sebastes diaconus*) found similar results. When removing the 92 identified SNPs presumably linked to sex, *F*_ST_ values between males and females decreased from 0.45 to 0.0036, although still significant (Vaux et al., 2019). Despite these results, little to no attention has been made to evaluate the importance of this type of marker in other species.

One specific application of genetic stock definition is to determine their relative contribution in a context of mixed stock fisheries. In particular, this issue may be addressed using assignment methods, which consist of determining the most probable origin (or reference stock in the case of fisheries) of an individual or a group of individuals from unknown sources based on multilocus genotype information (Beacham et al., 2017; Dann, Habicht, Baker, & Seeb, 2013; Ensing, Crozier, Boylan, O'Maoiléidigh, & McGinnity, 2013). The statistical power of assignment tests highly depends on the level of differentiation between putative source populations (Pritchard, Stephens, & Donnelly, 2000).

Greenland Halibut (*Reinhardtius hippoglossoides*), also named turbot, is a flatfish of the Pleuronectidae family with a circumpolar distribution throughout the Northern Hemisphere. In the Gulf of St. Lawrence (Canada), spawning occurs every year during the winter months from January to March. Following emergence, larvae drift for a few months and then settle in a nursery area until growth is complete (Sohn, Ciannelli, & Duffy‐Anderson, 2010). A nursery can be defined as an area where the density of juveniles (fish older than larvae, but in which gonads are not yet mature) is above average compared to elsewhere in the range of the species. These habitats are also usually characterized by an abundance of smaller prey and a relatively low predation rate (Beck et al., 2001; Dahlgren et al., 2006). There are two known nurseries of Greenland Halibut in the St. Lawrence system, one being located in the Estuary and the other in the Gulf north of Anticosti Island (Youcef, Lambert, & Audet, 2013). Benthopelagic fishes such as Greenland Halibut are characterized by a high potential to disperse due to the prolonged pelagic larval phase, which increases connectivity between populations, thus complicating the definition of genetically distinct population and management units (Bailey, 1997; Diopere et al., 2018; Hoarau, Rijnsdorp, Veer, Stam, & Olsen, 2002; Le Moan, Jiménez‐Mena, Bekkevold, & Hemmer‐Hansen, 2019; Vandamme et al., 2014). Several studies have aimed at documenting the extent of genetic connectivity of the species in the western Atlantic with different outcomes. First, several authors proposed that Greenland Halibut from the Gulf of St. Lawrence comprise a single population, distinct from other stocks in the Atlantic. This hypothesis has been supported from studies on allozymes (Fairbairn, 1981) and prevalence of parasites (Arthur & Albert, 1993; Khan, Dawe, Bowering, & Misra, 1982). Arthur and Albert (1993) looked more precisely within the Gulf of St. Lawrence and nearby Atlantic Ocean and further suggested that Halibut from the Saguenay Fjord was distinct from fish from the rest of St. Lawrence system. In contrast to those previous studies, Roy, Hardie, Treble, Reist, and Ruzzante (2014) suggested based on an analysis of 12 microsatellite markers suggested that Greenland Halibut from that St. Lawrence system, eastern Labrador, and Newfoundland comprised a single panmictic population (Roy et al., 2014). It is possible that these contradictory results stem from the limited resolution offered by previous types and number of genetic markers that were used previously to assess population structure in Greenland Halibut. This situation supports revisiting the population genetics of the species using a more powerful method, such as genotyping‐by‐sequencing (GBS). Moreover, no study has attempted to document the contribution of different putative Halibut populations to the juveniles that use the two known nurseries within the St. Lawrence system.

The first goal of this study was to test the null hypothesis of the absence of population structure among Greenland Halibut from the St. Lawrence system and Atlantic sampling locations near Newfoundland. The second goal was to determine the origin of juveniles from the two nurseries located within the St. Lawrence system. We used GBS to genotype a total of 850 adults from 7 localities distributed from Saguenay Fjord to offshore of Newfoundland and 200 juveniles from both nurseries (two annual temporal replicates of 100 for each nursery). In addition, we assessed the effect of sex‐linked markers on the definition of population structure. In light of our results, we discuss (a) the importance of using a large number of markers to refine the resolution of population structure in weakly structured marine species, (b) the importance of sexing individuals being genotyped, and (c) the importance of performing temporal replicates to assess the temporal stability of patterns being documented.

## 2 MATERIAL AND METHODS

### 2.1 Sampling

**FIGURE 1:**
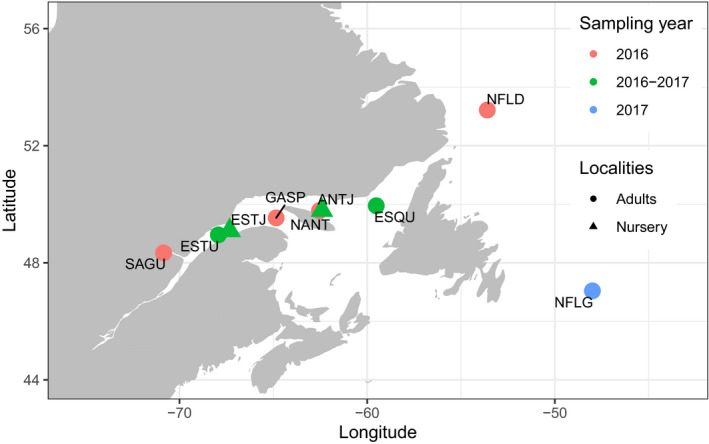
Map of sampling sites in Saguenay Fjord (SAGU), St. Lawrence Estuary (ESTU, ESTJ), Gaspé (GASP), North Anticosti Island (NANT, ANTJ), Esquiman (ESQU), and Atlantic Ocean (NFLD, NFLG). The Estuary and northern Anticosti Island nurseries (ESTJ and ANTJ) are defined by the triangles and adult populations of the St. Lawrence and Atlantic (SAGU, NFLD, ESTU, NANT, GASP, and ESQU) by circles. Sites that were sampled for a single year are colored in pink for the first year of sampling and in blue for the second. Those sampled for the two consecutive years are colored in green

Fisheries and Oceans Canada (DFO) and volunteer fishermen from the area collaborated to achieve the sampling (Figure 1). Every fish was measured and its sex identified at the time of capture. Fish larger than 31 cm were considered mature, whereas those under were classified as juveniles (DFO, 2012; Morin and Bernier, 2003). The nurseries sampled in the St. Lawrence are located in the estuary (ESTJ; 49.0993, −67.3247) and the North of Anticosti Island (ANTJ; 49.7913, −62.4102). A total of 100 individuals per year from each nursery were collected in late summer of 2016 and 2017, totaling 400 juvenile samples. We also sampled seven adult localities, namely Saguenay (SAGU; 48.339722, −70,849.722), Estuary (ESTU; 48.9548, −67.9288), northern Anticosti Island (NANT; 49.7807, −62.5422), Gaspé (GASP; 49.533333, −64.85), Esquiman (ESQU; 49.9485, −59.516), Labrador (NFLD; 53.218056, −53.600556), and Newfoundland (NFLG; 47.04, −48). Presumed adult stocks of Greenland Halibut were selected based on their proximity to the two nurseries and to provide longitudinal coverage of its range in the St. Lawrence system, with the assumption that this stock might be partially or completely isolated. We also included two sampling sites outside the St. Lawrence to test whether the Atlantic stock is genetically differentiated to the St. Lawrence region. Fifty adult individuals for each site, with ESTA, ESQU sampled twice, that is late summer of 2016 and 2017 (total of 450 adult samples). Part of the pelvic fin was recovered and stored in 95% ethanol (EtOH) until DNA extraction.

### 2.2 DNA extraction

The genetic material was extracted according to the protocol detailed in Aljanabi and Martinez (1997). Samples were migrated on a 1% agarose gel to ensure genomic DNA was sufficiently abundant and of high quality for DNA sequencing. DNA concentration was determined by colorimetry using a spectrophotometer (NanoDrop).

### 2.3 Bioinformatics and genotyping

Following DNA extraction, every sample was diluted to a concentration of 10 ng/μl in 10 μl to normalize at 100 ng of DNA per individual. A total of ten 96‐pools chips with a mean of 75 individuals were sequenced, for a total of 10 libraries. Double‐digest RAD‐sequencing libraries were prepared using a protocol inspired by Mascher et al. (2013) and Abed et al. (2019). Two nonspecific endonucleases (namely *PstI* and *MspI*) and a digestion buffer (NEB4) were combined to cut genomic DNA for a period of two hours at 37°C. Chips were then incubated at 65°C for 20 min to stop enzymatic reaction. DNA fragments were combined to adaptors at both ends using a ligation master mix and a T4 ligase. All samples were incubated at 22°C for 2 hr. Prepared DNA fragments were pooled in a 96‐plex and cleaned using a QIAquick PCR purification kit. Every sampled individual was barcoded with a unique six nucleotide sequence. After the amplification of pooled fragments was completed using PCR, a test for quality control was done using a BioAnalyser. Sequences retained for sequencing range from 150 to 350 bp. Samples were sequenced on the platform ION PROTONTM System (Life Technologies) located at the Institut de Biologie Intégrative et des Systèmes (Laval University).

The resulting dataset was checked for overall quality using FASTQC (Andrews, 2010). Adapters were removed from reads using cutadapt v.1.8.1 (Martin, 2011). Sequences were demultiplexed and truncated to 80 bp with the process_radtags module implemented in the software STACKS v1.48 (Catchen, Hohenlohe, Bassham, Amores, & Cresko, 2013). Cleaned DNA reads were aligned to a mostly complete (L50 = 16 scaffolds, N50 = 17.64 Mb) reference genome of a mature female Halibut from the Estuary of St. Lawrence that was assembled at McGill University and Genome Quebec Innovation Center (Ferchaud et al., unpublished data) with the package software BWA (Li & Durbin, 2009) with parameters fixed at 500 for the maximum occurrence of a read (c = 500), no gap open penalty (O = 0), a gap extension penalty of 2.2 (E = 2.2), and alignments with score lower than 0 were discarded (T = 0). SNPs were identified at each locus with pstacks with a minimum depth of coverage to report a stack set to 1 (*m* = 1). Then, cstacks was selected to construct a reference catalog with default parameters using all the individuals sampled in adult localities and nurseries. Individual SNPs were called using the catalog with sstacks also with parameter left as is. The minimum coverage depth for a locus was fixed to 7 (*m* = 7) using populations module in STACKS set with the minimum percentage of individuals in a population required to process a locus set to 60% (*r* = .6), the minimum number of populations a locus must be present to process it fixed at 6 (*p* = 6, while the population map included the seven sample localities plus the two nurseries) and the maximum *p*‐value to keep a *F*_ST_ measurement set to 0.1. After, the ddRADseq dataset was filtered for SNPs calling at over 70% of genotyped individuals in every population (see details in Table 1). Following filtering was performed with stacks_workflow available online (https://github.com/enormandeau/stacks_workflow). Reads were filtered for a minimum coverage depth of 10× and a maximum coverage depth of 100×. This step ensures working with markers that have enough coverage to limit false positives and also remove those that could be located in repetitive segments in the genome, which causes SNP overrepresentation. Next, we removed SNPs that were genotyped in fewer than 70% of overall individuals. Observed heterozygosity was set to be superior than 0.5 (Ho > 0.5) within samples in order to remove potential homeologs from the dataset (Davey et al., 2011). Both individuals and loci with a proportion of more than 10% of missing genotype overall were discarded. Minor allele frequency was set to more than 0.001 (MAF > 0.001) for all of the sampling sites to make sure any sequencing error from the dataset was removed. Finally, a relatedness analysis was performed to remove individuals closely related or that may have been pooled twice (relatedness > 0.9) using VCFtools (Danecek et al., 2011). When a pair of individuals with a high relatedness coefficient originated from the same sampling site, one was selected randomly to be kept in the dataset, if they were from different sites, both individuals were discarded. One SNP per read was kept based on which one had the highest minor allele frequency. We performed this type of filtering selection in order to increasing discrimination power needed to detect differentiation with a marine species (Gagnaire et al., 2015).

**Table 1:** Number and percentage of remaining loci after every filtering step

Filter	Number of remaining individuals	Number of remaining loci	% of remaining loci	% of remaining loci
No filter	752	317,173	100.0	100.0
Missing data	720	—	100.0	100.0
Global MAF > 0.001	—	228,603	72.1	72.1
Genotyped individuals > 0.7	—	57,753	18.2	18.2
Relatedness	688	—	18.2	18.2
Heterozygosity > 0.6	—	56,129	17.7	17.7
Sex‐related SNPs	—	55,693	17.6	17.6
1 SNP/locus	—	32,193	10.1	10.1
Genome scan		26,681	8.5	8.5

### 2.4 Identifying sex‐linked and selected SNPs

We then removed SNPs under balanced selection using Bayescan v.2.1 (Foll & Gaggiotti, 2008). Balancing selection can take different forms, including disassortative mating, frequency‐dependent selection, overdominance, or selection that varies in direction and intensity across space or time. Any of these processes will generally result in higher allelic diversity and/or less population differentiation at loci under balancing selection (King, Stansfield, & Mulligan, 2006). This software uses a Bayesian approach to estimate *F*_ST_ coefficients. It categorizes SNPs under balanced and directional selection based on a posterior distribution set by the user, which determines how the neutral model is more likely to occur. We used a posterior odd of 10,000, as suggested by Lotterhos and Whitlock (2015), with 9,999 pairwise iterations. For further analysis, we kept all neutral and divergent loci, to keep all variation explained by either reproductive isolation or differentiation in selection pressure between sites (Gagnaire et al., 2015). We chose to remove markers potentially under balancing selection since this force causes both alleles to be maintained equally frequent when comparing sites, which reduce discrimination power and may wrongly lead to overinterpreting the extent of genetic connectivity between populations (e.g., Benestan et al., 2015).

After removing SNPs under balancing selection, the dataset was duplicated. One duplicate set was left as is and the other was used to remove sex‐linked markers. To identify SNPs associated with sex, we performed a redundancy analysis (RDA) using the R package vegan (Oksanen et al., 2007). The dependant matrix was represented by the genotypic data and the explanatory variable as sex. A SNP was correlated with the sex variable when the standard deviation for the distribution of RDA loadings was higher than 95%. To perform following analysis, the VCF file obtained from the filtering steps was converted to a genepop file using PGDspider (Lischer & Excoffier, 2012), as well as VCFtools to translate into a PLINK file (Purcell et al., 2007).

### 2.5 Population structure

Pairwise *F*_ST_ (Wright, 1931) estimations between sampling (including nurseries) sites with and without sex‐linked SNPs were done using the software GenoDive v.3.0 (Meirmans & Van Tienderen, 2004), testing for 9,999 permutations to estimate *p*‐values. To investigate clustering for both datasets, we performed principal component analysis (PCA) using adegenet v.2.0.0 (Jombart, 2008) using the R software v.1.1.442. Thereafter, ADMIXTURE v.1.23 software (Alexander, Novembre, & Lange, 2009) was used to infer the membership of an individual and its probability of belonging to a putative ancestral population by comparing pairwise genetic differentiation (*F*_ST_) at every SNP. We used cluster values (*K*) ranging from 1 to 10 to test for the lowest value of cross‐validation error. We used 10,000 permutations and other parameters (random seed, block relaxation algorithm, termination criterion at *ε* = 10^–4^, quasi‐Newton convergence acceleration method, and bootstrap replicates set to 200) were left as is. Population structure was investigated for the dataset with and without sex‐linked markers previously identified. A Mantel test between *F*_ST _estimates of both SNPs datasets was performed to estimate possible biases caused by sex‐linked markers on population structure using the vegan package (Oksanen et al., 2007), using the Spearman method and 9,999 permutations.

### 2.6 Assignment of juveniles

We performed an individual assignment test with the R package assignPOP v.1.1.7 (Chen et al., 2018) using only the dataset without sex‐linked markers. We used the function assign.X set to the support vector machine (SVM) model to distribute juveniles in reference populations defined in the previous step. A function implemented in this package performs a cross‐validation error using Markov chain Monte Carlo (MCMC) by reassigning individuals of known origin (i.e., reference individuals) to determine statistical accuracy for the membership of unknown individuals. We tested for three proportions of individuals in training group (0.5, 0.7, and 0.9) and five different proportions of loci ranked in decreasing *F*_ST_ values (0.1, 0.25, 0.5, 0.75, and 1) to test whether an increase of loci result in an increased assignment precision. To test for differences of contribution from the different stocks between nurseries, we performed chi‐squared tests between proportion of juveniles from each nursery assigned to reference stocks for both years of sampling, for a total of four tests (first year/ANTJ, first year/ESTJ, second year/ANTJ, and second year/ESTJ).

## 3 RESULTS

### 3.1 Genotype‐by‐sequencing

The mean read coverage depth was 50× overall. For additional details about general quality of the data, see Figures S1 and S2. In total, 32 individuals were removed because of high mean missing data and another 32 individuals because of high relatedness (>0.9). All of the subsequent analyses were made using a total of 26,965 SNPs when sex‐linked SNPs were kept and 26,681 SNPs when they were removed (see below and Table 1 for details).

### 3.2 Selection of SNPs

Out of 55,993 filtered SNPs, the selection of one SNP per locus based on the highest minor allele frequency narrowed the dataset to 32,192 markers. Results from the genome scan show the most markers under selection were under balancing selection (5,294 SNPs, 16.4% of dataset versus directional selection at 15 SNPs, 0.05% of dataset). Putative neutral markers represented 82.8% of dataset (26,669 SNPs). The overall RDA was significant (*p*‐value = .001). When looking at axes separately, the first axis of RDA explained 99.9% of observed genetic variance, corresponding to an eigenvalue of 116.54 and a *p*‐value of .001 (see Figure S3). A total of 436 SNPs were identified as sex‐biased. Those SNPs were located primarily on two scaffolds (one gathering 205 and the other 140), thus supporting the identification of sex chromosomes for this species. This result is consistent with the linkage map of Atlantic Halibut (*Hippoglossus hipoglossus*), the most closely related species to Greenland Halibut (Reid et al., 2007). Out of the 850 samples, 98 individuals distributed across all sampling sites were not sequenced because of the lack of quality of extracted DNA. On average, there were about 2,000,000 sequenced reads/sample for a total of 10 pooled libraries.

### 3.3 F‐statistics

Pairwise *F*_ST _values were calculated between all sampling sites (including nurseries) were relatively low (see Table 2). For the dataset including sex‐linked markers, we found a significant difference between fish originating from the Atlantic Ocean with those of North of Anticosti Island and all locations in the St. Lawrence system (*p*‐value < .05). This result slightly contrasts with the dataset excluding sex‐linked markers. Thus, we found no significant genetic difference between all sampling sites (including North of Anticosti Island) in the Estuary and the Gulf of St‐Lawrence (*p*‐value > .05). However, the two sites offshore of Newfoundland were significantly distinct from all sites within the St. Lawrence system, which support the hypothesis that these two regions comprise genetically distinct stocks (global *F*_ST_ when combining sites = 0.00146, *p*‐value = .001). While such *F*_ST_ value appears low, it is nevertheless typical of values reported in other marine species on a similar geographical scale (e.g., Benestan et al., 2015; Xuereb et al., 2018).

**Table 2:** Pairwise differentiation index (*F*_ST_) of Greenland Halibut from seven sampling sites distributed from the Saguenay Fjord to offshore Newfoundland

	SAGU	ESTU	GASP	NANT	ESQU	NFLD	NFLG
**SAGU**	—	0.00006	0.00029	0.00031	0.00045	0.00221*	0.00218*
**ESTU**	0.00016	—	0.00041	0.00104*	0.00024	0.00153*	0.00180*
**GASP**	0.00029	0.00040	—	0.00124*	0.00092	0.00246*	0.00283*
**NANT**	0.00031	0.00023	0.00044	—	0.00012	0.00157*	0.00189*
**ESQU**	0.00015	0.00010	0.00046	0.00012	—	0.00153*	0.00151*
**NFLD**	0.00194*	0.00142*	0.00206*	0.00150*	0.00146*	—	0.00025
**NFLG**	0.00192*	0.00176*	0.00255*	0.00188*	0.00156*	0.00043	—

Note

Sites ESTU and ESQU were sampled for two consecutive years of sampling. Values above the main diagonal represent the dataset with sex‐linked markers and below the dataset without those markers. Significant *F*_ST_ (*p*‐value < .05) is marked with an asterisk.

### 3.4 Effect of sex‐linked markers on population structure

**FIGURE 2:**
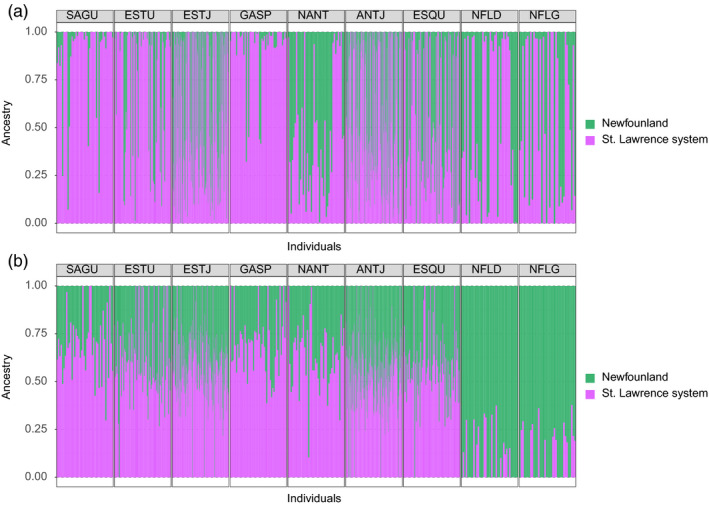
Population structure for Greenland Halibut from the Estuary/Gulf of St. Lawrence (Saguenay Fjord SAGU, St. Lawrence Estuary ESTU, St. Lawrence Estuary nursery ESTJ, Gaspé GASP, North Anticosti Island NANT, North Anticosti Island nursery ANTJ, and Esquiman ESQU) and Newfoundland (NFLD and NFLG) using ADMIXTURE: (a) for dataset including sex‐linked markers and (b) for dataset without sex‐linked markers. Results are shown for *K* = 2. Sites are classified from west to east and individuals in alphanumerical order. Sites sampled for two consecutive years are combined

**FIGURE 3:**
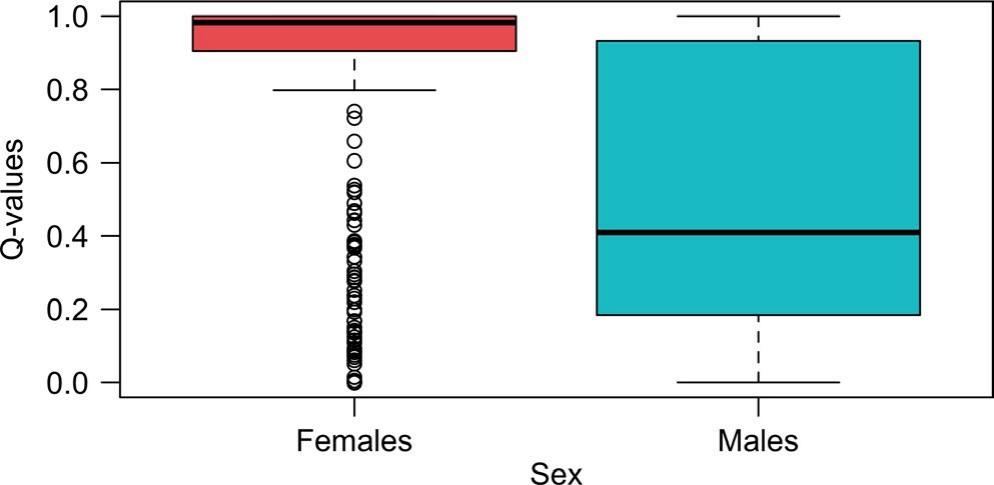
Boxplot of *q*‐values separated by sex when performing ADMIXTURE using the dataset with sex‐linked markers

**FIGURE 4:**
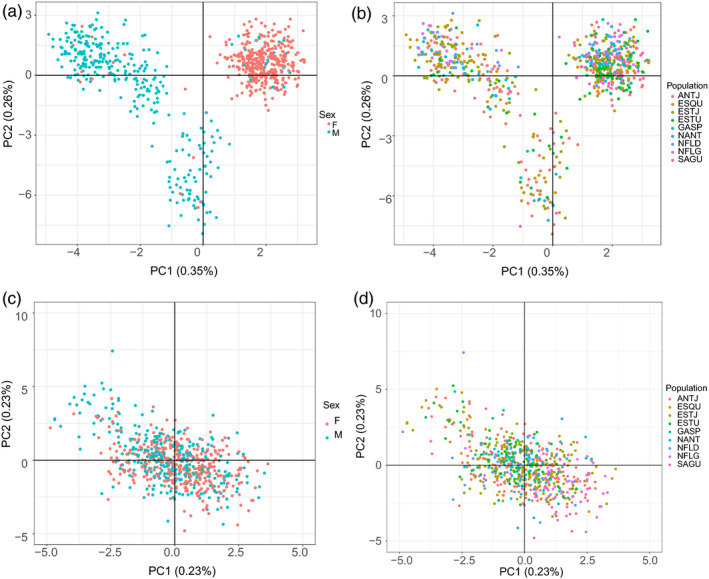
Principal component analysis (PCA) using (a) and (b) dataset including sex‐linked markers and (c) and (d) the dataset without sex‐linked markers. Females are colored in pink and males in blue in (a) and (c) and individuals are colored by population in (b) and (d)

Concerning the clustering analysis, the lowest cross‐validation error was obtained when grouping individuals into one cluster for datasets with and without sex‐linked markers (see Figure [Link], [Link]). However, since *F*‐statistics revealed a significant difference between the Gulf of St. Lawrence and the Atlantic sites outside the Gulf, we decided to investigate results for *K* = 2. For the dataset with sex‐linked SNPs, the ADMIXTURE plot revealed no geographical trend in population structure, with most individuals clustering into two distinct groups corresponding to males and females (Figure 2a). Both groups can also be visualized in the principal component analysis with a few individuals clustering with the opposite sex (Figure 4a) and in the boxplot of *q*‐values (see Figure 3). Individuals with admixture coefficient most similar with the opposite sex from which they were identified correspond mostly to young juveniles, for which the visual sexing can be challenging and possibly leading to mistakes. Although these individuals represented an important part of the dataset, removing them before identifying sex‐linked markers did not result in a significantly different list of SNPs. For the population structure for the dataset without sex‐linked SNPs, the ancestry analysis showed a segregation of adult halibuts between the Gulf of St‐Lawrence and the Atlantic Ocean (Figure 2b). This result was supported by the pairwise *F*_ST_ estimates between sampling sites without sex‐linked markers (Table 2). Despite this significant differentiation between both regions, some individuals collected within the Gulf of St. Lawrence seemed to be more strongly associated with the population of Newfoundland (ESTU and ESQU; *q*‐values ranging from 1e−05 to 0.450 for ESQU and from 0.014 to 0.490 for ESTU). This observation, with the absence of “pure” individuals, adds weight to the occurrence of genetic (and demographic) connectivity between these two stocks despite significant differentiation. Gene flow seems to be asymmetric between both stocks, since admixed individuals are predominantly present in the St. Lawrence system instead of the Atlantic. This observation suggests a higher dispersion rate from the ocean toward the Gulf than in the opposite direction.

### 3.5 Assignment of juveniles to identified stocks

**FIGURE 5:**
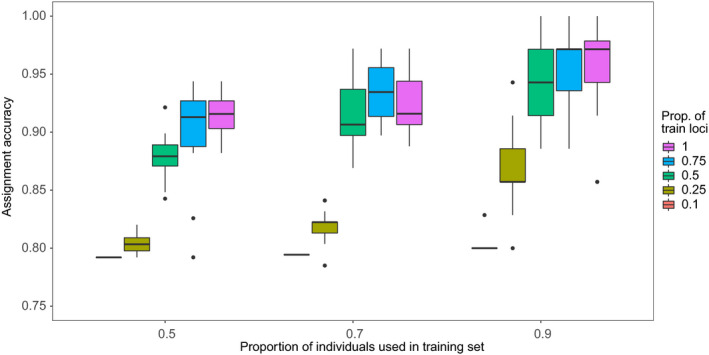
Monte Carlo cross‐validation test for SNPs dataset using different proportions of training loci (0.1, 0.25, 0.5, 0.75, and 1) and individuals in training sets. Boxplots were generated for overall sources populations (left) and for both populations separately (GSL in center and NFL to the right). Selected SNPs were ranked in decreasing values of differentiation index when using a different proportion then 1

**FIGURE 6:**
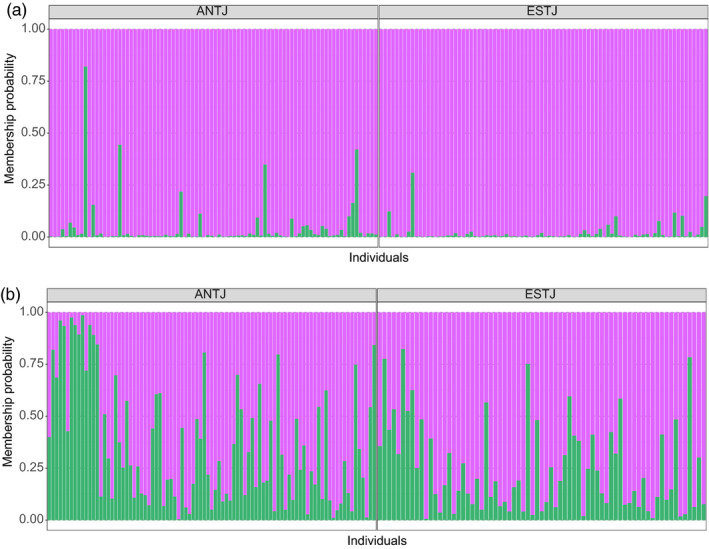
Individual assignment test (a) for the first year of sampling and (b) for the second year of sampling to reference stocks of Gulf of St. Lawrence (GSL) and Newfoundland (NFL). All loci and individuals were kept for the test

For all assignment tests, the reference populations (sample sites without the nurseries) corresponded to the clusters identified in the dataset without sex‐linked markers, namely the Gulf of St. Lawrence (GSL) and Newfoundland (NFL). Monte Carlo cross‐validation test showed an improvement of correct assignment for both reference stocks when including all loci (Figure 5). For this reason, we kept all markers for subsequent analysis. Difference between the corresponding proportion of juveniles between years was significant (*p*‐value = 6.882e−07 for ANTJ and *p*‐value = .001964 for ESTJ). Also, for the second year of sampling, proportion of juveniles originating from NFL were higher for the ANTJ nursery than ESTJ (.3034 vs. .1389, *p*‐value = .04775). Therefore, this corresponded to an average juvenile contribution of the Newfoundland stock during the second year of sampling, albeit with a higher proportion of juveniles in the most eastern ANTJ nursery (30.33% for ANTJ and 13.89% for ESTJ; Figure 6).

## 4 DISCUSSION

The main purposes of this study were to determine the origin of Greenland Halibut juveniles from nurseries within the St. Lawrence system and to test for the relative contribution of the different stocks. First, we performed a structure analysis with ADMIXTURE and a PCA to identify adult populations to be used as reference for the population assignment with SNPs defined by GBS. We found that sex‐linked markers affect significantly structure result by masking signal of differentiation between the GSL and NFL stocks that were defined. Second, we used the software assignPOP to determine the most likely stock of origin of juveniles of two different years of sampling between populations identified previously without sex‐linked markers. Our results revealed an important inter‐annual difference in proportion of juveniles originating from the NFL population, from being almost absent in the first year to almost a third of the total contribution the second year. We discuss the implications of these results on management strategies.

### 4.1 Population structure: effect of sex‐linked markers

Our results show a significant differentiation between geographical regions of Atlantic Ocean and the St. Lawrence, including the Saguenay Fjord, the Estuary, and the Gulf (*p*‐value = .001) but no evidence of population structure within St. Lawrence system with the dataset. This result is similar to the analysis of allozymes done by Fairbairn (1981), which supported a pattern of high gene flow between the St. Lawrence and the Atlantic Ocean, although St. Lawrence showed a significant signal of isolation based on two unique allozymes found in this region. Still, it is probable that the significant pairwise *F*_ST_ value we observed might be a consequence of the increased number of loci and samples, which inflates chances of obtaining false positive, instead of being a biological genomic differentiation signal (Helyar et al., 2011). On the other hand, as mentioned above, weak but biologically significant values of genetic differentiation have frequently been reported in the literature for marine species (e.g., Benestan et al., 2015; Xuereb et al., 2018). Here, the biological significance is revealed by the fact that we could confidently reassign juvenile fish from the nurseries to one population or the other, in proportion varying between years. This would not be possible if the genomic differentiation had not biological meaning.

Here, we showed that population structure was different when removing sex‐linked SNPs from dataset. This result corroborates the study of Benestan et al. (2017) who showed that 12 highly differentiated sex‐associated SNPs (with a total of approximately 10,000 SNPs) were sufficient to create population structure previously found between inshore and offshore locations in American lobster (*Homarus americanus*). They are also similar to results obtained with deacon rockfish (*Sebastes diaconus*) in Vaux et al. (2019), where a less distinct population structure was found between male and female when removing the 92 SNPs associated with sex. Our results add weight to these findings and show the importance of considering sex‐linked markers in population genomics studies for species, especially in species characterized by weak genetic differentiation. To our knowledge, only a few studies have either focused on or identified sex‐related SNPs to remove them from dataset since the original publication (Bissegger, Laurentino, Roesti, & Berner, 2019; Devloo‐Delva et al., 2019; Grummer et al., 2019; Jackson, Roegner, & O'Malley, 2018; Lemopoulos et al., 2019; Messmer et al., 2018; Ogata, Lambert, Ezaz, & Miura, 2018; Vaux et al., 2019).

Another salient result of our study is that current management strategy in the southwestern distribution range of Greenland Halibut appears to reflect real population structure. To be more accurate, our results suggest that management zones 4R, 4S, and 4T should be combined and managed as single unit, which is already the case (DFO, 2018). Even though no sample sites were located in the 4T zone, our findings show that there is no sign indicating a significant differentiation from the rest of the Gulf based on population genomics. Admittedly however, other factors pertaining to fisheries management could justify dividing genetically identified populations into subunits, such as fish landings. For instance, since 2012, DFO surveys gathering information on landings from fishermen and their own data found a regional difference in abundance between the North of Anticosti Island and the west of the Gulf, where a decrease of approximately 350 t. in landing has been reported (DFO, 2015).

### 4.2 Assignment of juveniles to identified stocks

The two identified stocks (St. Lawrence and Newfoundland) served as reference populations to assign unknown juveniles from the Estuary and the North of Anticosti nurseries. Our finding shows that proportion of juveniles varies significantly between years. For the first year of sampling, stock of Gulf of St. Lawrence contributes entirely to the juvenile populations in both nurseries. However, contribution of the Newfoundland stock represents almost a third of juveniles the following year, with a slightly higher proportion of juveniles in the ANTJ nursery (30.33% for ANTJ and 13.89% for ESTJ). This demonstrates that recruitment within the St. Lawrence system depends mainly on the Gulf of St. Lawrence stock. This then also raises the hypothesis of the existence of other nurseries that would be mainly used by the Newfoundland stock within the Atlantic Ocean that still remain to be found. For instance, one such possible location could be Disko Bay, which has been suspected (but not confirmed) to be the main nursery for Greenland Halibut in the east of Atlantic Ocean population (Cooper, Maslenikov, & Gunderson, 2007; Junquera, Roman, Morgan, Sainza, & Ramilo, 2003; Riget & Boje, 1989). This would not necessarily exclude that the Gulf of St. Lawrence stock could also contribute to this nursery. For instance, fish from the St. Lawrence system could potentially drift during their prolonged pelagic larval phase and enter via the Belle‐Isle strait, where there is a cold‐water current oriented toward the Gulf coming from Labrador (Han, Loder, & Smith, 1999; Saucier, Roy, Gilbert, Pellerin, & Ritchie, 2003; Wu, Tang, & Hannah, 2012). We also hypothesize that the proportion of larvae entering the Gulf might depend on the strength of this current during the same year, which would need to be more formally tested. This could also explain why a significant superior number of juveniles from the Atlantic are found in the more eastern ANTJ nursery. Belle‐Isle strait has been reported to be an important migration route for several species, including Atlantic redfish (*Sebastes mentalla*; Benestan et al., unpublished data), snow crab (*Chionoecetes opilio*; Puebla et al., 2008), and capelin (*Mallotus villosus*; Cayuela et al., 2019; Kenchington, Nakashima, Taggart, & Hamilton, 2015). More specifically, the study on redfish aimed at documenting the connectivity between two ecotypes: deep water ecotype, found in Gulf of St. Lawrence, and the shallow water ecotype, found mostly in Labrador Sea and eastern Newfoundland coast. Authors found more than 10% of the total number of individuals classified as the GSL ecotype in the region corresponding to the shallow ecotype. From this finding, linked to the low density of adult located in the Belle‐Isle strait, the authors suggested that larval drift plays a role in gene flow between the two ecotypes. For management purposes, the inter‐annual variation shown in population assignment demonstrates the relevance of performing temporal replicates to better reflect the biological dynamics of the system. As such, our results also show that for questions related to recruitment, time needs to be taken into account even for population of large size such as Greenland Halibut. Even though individual assignment is a reliable method to determine the origin of unknown individuals using frequentist statistics, we were not able to corroborate these results using a likelihood method (i.e., genetic stock identification). Assignment output from software gsi_sim (Anderson, Waples, & Kalinowski, 2008) classified all juveniles from both years of sampling to the GSL stock without mixture, which does not represent the extant of genetic differentiation revealed by significant *F*_ST_ values. This method of assignment has also proven to be useful for fisheries management of multiple species, including Chinook salmon (Beacham et al., 2017; Satterthwaite et al., 2015), Atlantic salmon (Griffiths et al., 2010), and Green sturgeon (Israel et al., 2009). Clearly, although significant, the extant of population differentiation measured here is most likely too low to efficiently use this approach.

### 4.3 Conclusion and perspective

Overall, our results show that performing both sex identification and temporal replicates are important in the type of study we report here, especially so when studying weakly structured marine species. This study is also part of a larger ongoing research program on Greenland Halibut which overall aims at contributing to the improvement of management of exploited stocks of Greenland Halibut in Canada. More specifically, data are being collected from sampling sites representing the whole western Atlantic range limit of this species in order to improve our understanding of genomic connectivity within the Atlantic Ocean using whole‐genome resequencing, documenting migration pattern of juveniles by means of otolith chemistry analysis, and evaluating the effect of sex and temperature on juvenile growth (Ghinter, Lambert, & Audet, 2019). Finally, our study revealed the absence of significant population structure within the St. Lawrence system despite the occurrence of a pronounced gradient in environmental conditions in this system (DFO, 2013). Given this result and also is the increasing number of studies have revealed epigenetic structure among weakly genetically differentiated populations in the wild (e.g., Johnson & Kelly, 2019; Keller et al., 2016; Wenzel et al., 2014; Zoldos et al., 2018), it would be relevant in future studies to investigate patterns of methylation between Greenland Halibut from different locations characterized by distinct environmental features.

## CONFLICT OF INTEREST

None declared.

## Supporting information

Figure S1

Figure S2

Figure S3

Figure S4A

Figure S4B

## Data Availability

Raw demultiplexed sequences are available on the Sequence Read Archive (SRA) on the study accession number: PRJNA613476.
