# Supplementary figures and images for: Estimating the contribution of Greenland Halibut (Reinhardtius hippoglossoides) stocks to nurseries by means of genotyping‐by‐sequencing: Sex and time matter
Source: Evol Appl. 2020 May 20;13(9):2155–67. doi: 10.1111/eva.12979 (PMC7513701; doi:10.1111/eva.12979)

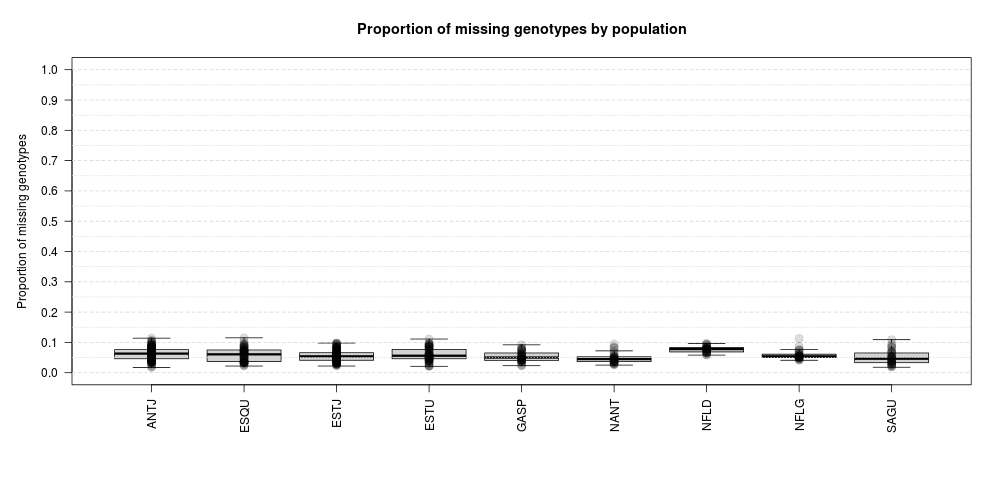

Supplement: Supplementary file 1 — Figure S1 [file EVA-13-2155-s001.png]

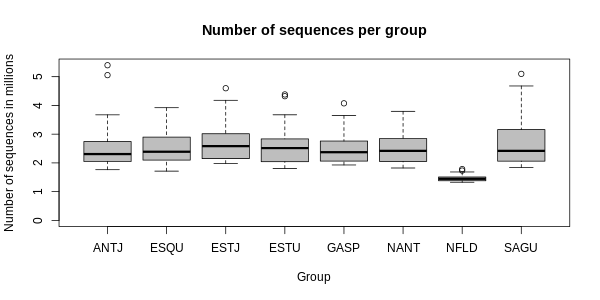

Supplement: Supplementary file 2 — Figure S2 [file EVA-13-2155-s002.png]

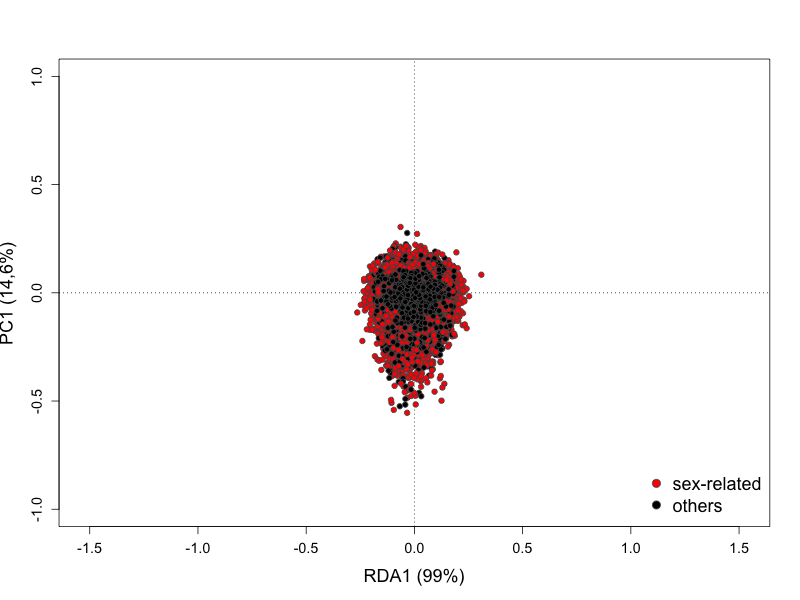

Supplement: Supplementary file 3 — Figure S3 [file EVA-13-2155-s003.png]

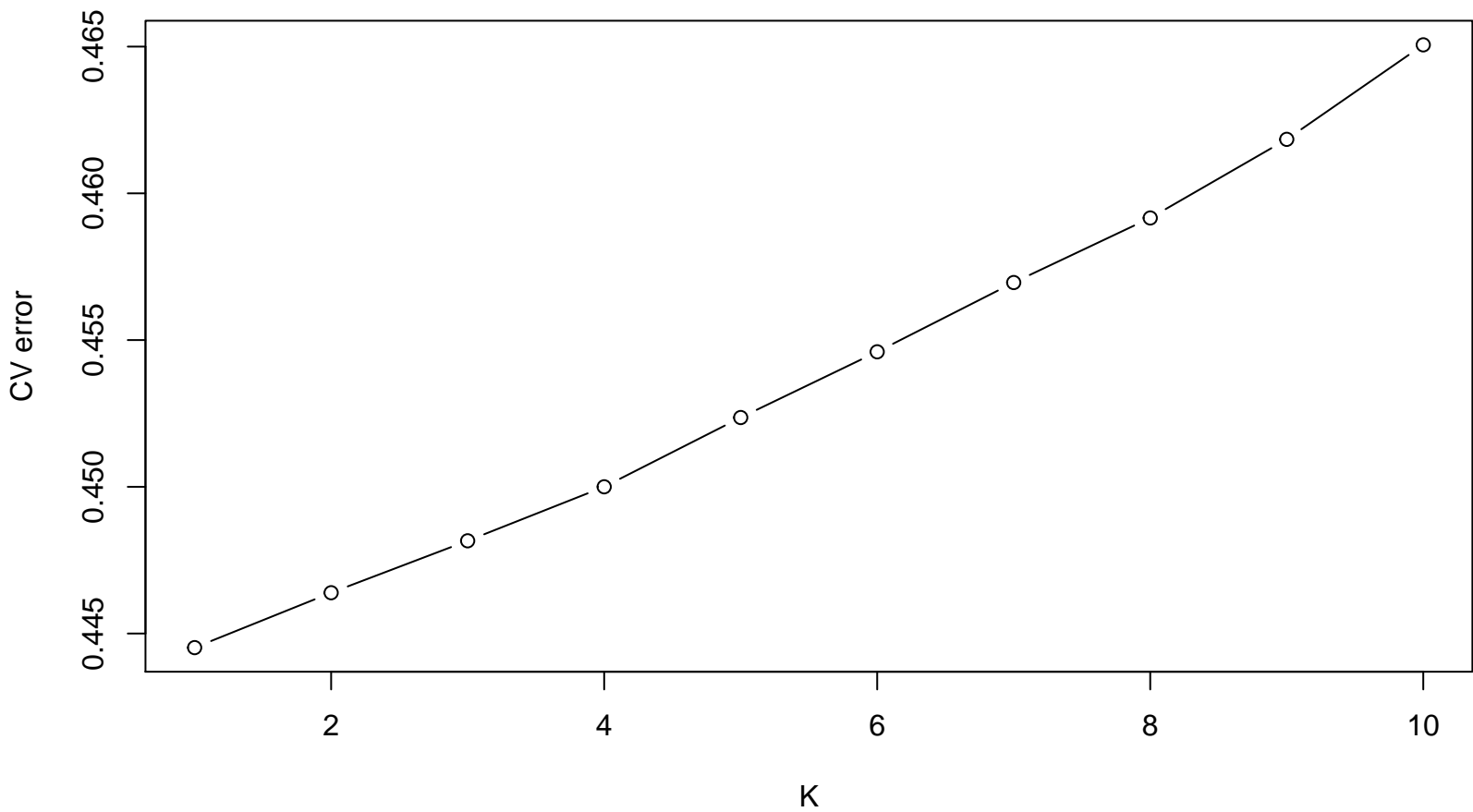

Supplement: Supplementary file 4 — Figure S4A [file EVA-13-2155-s004.pdf]

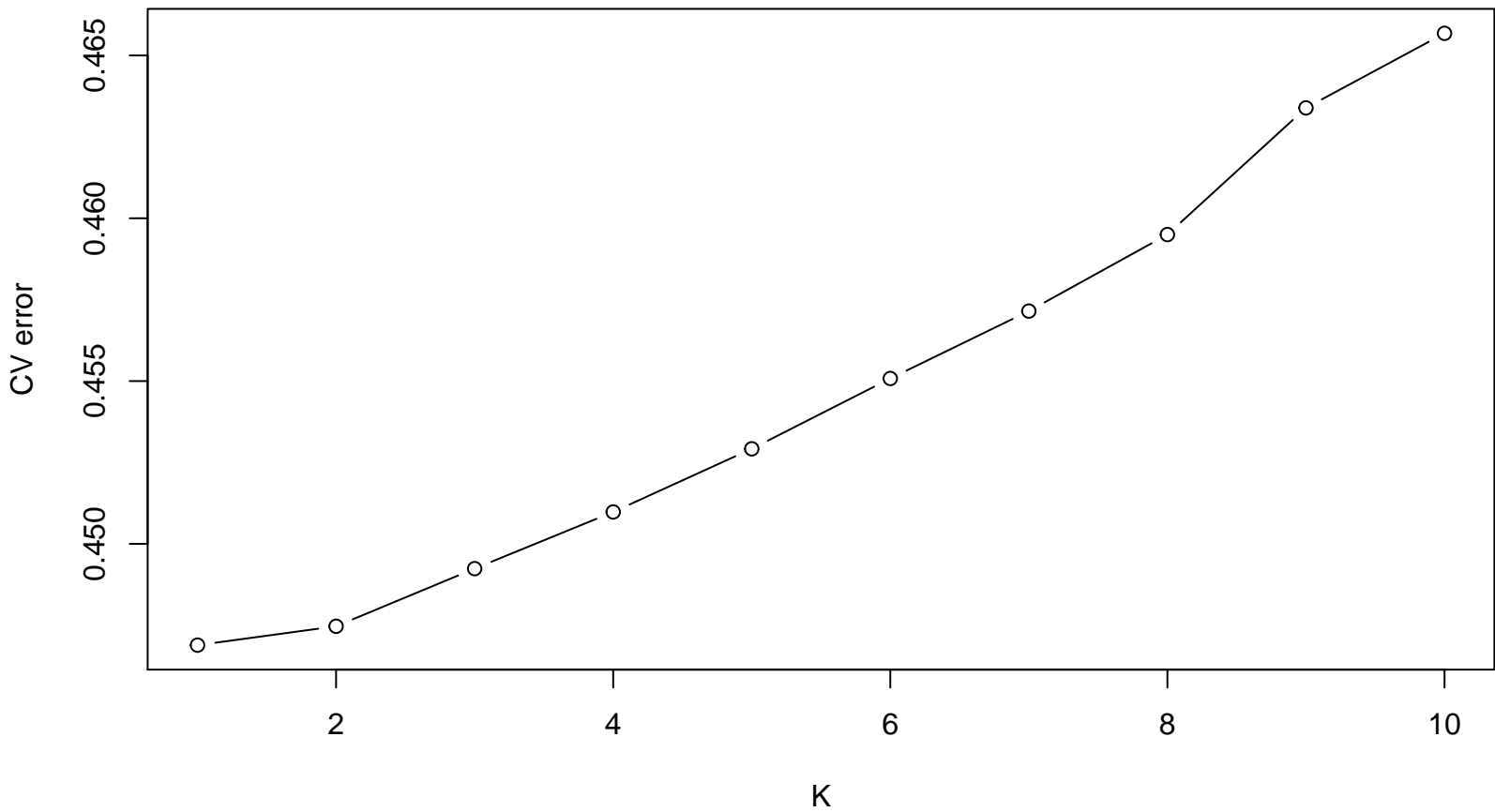

Supplement: Supplementary file 5 — Figure S4B [file EVA-13-2155-s005.pdf]
